# Analysis of variation of serum CEA, SCC, CYFRA21-1 in patients with lung cancer and their diagnostic value with EBUS-TBNA

**DOI:** 10.5937/jomb0-37083

**Published:** 2024-06-15

**Authors:** Yanjia Du, Ya Wen, Jieyu Huang

**Affiliations:** 1 Meizhou Peopležs Hospital, Department of Respiratory and Critical Care Medicine, Meizhou City, China

**Keywords:** lung cancer, carcinoembryonic antigen, cytokeratin 19 fragment, squamous cell carcinoma antigen, ultrasound-guided transbronchial needle aspiration, joint diagnosis, diagnostic value, pathological type, karcinom pluća, karcinoembrionalni antigen, citokeratin 19 fragment, antigen skvamoznih ćelija, ultra zvučno vođena transbronhijalna aspiracija iglom, zajednička dijagnoza, dijagnostički značaj, patološki tip

## Abstract

**Background:**

To explore the variation of serum carcinoembryonic antigen (CEA), cytokeratin 19 fragment (CYFRA21-1), and squamous cell carcinoma (SCC) antigen in patients with lung cancer (LC) and their diagnostic value with endobronchial ultrasound-guided transbronchial needle aspiration (EBUS-TBNA).

**Methods:**

This study examined the diagnostic value of serum tumor marker testing and EBUS-TBNA joint detection for LC in 150 patients with suspected LC.

**Results:**

Compared to benign patients, the serum levels of CYFRA21-1, SCC, and CEA in LC were higher (P<0.05). In patients with squamous cell carcinoma (LSCC), small cell lung cancer (SCLC), and lung adenocarcinoma, lung adenocarcinoma had higher serum CEA levels (P<0.05). In comparison, LSCC patients had higher serum SCC and CYFRA21-1 levels (P<0.05). As compared to each index detected alone, the AUC of combined detection of each index to diagnose LC and identify pathological types of LC was elevated.

**Conclusions:**

The clinical significance of serum CYFRA21-1, SCC, and CEA conjugated with EBUS-TBNA is demonstrated for diagnostic purposes and identification of LC pathological types.

## Abstract (sr)

**Uvod:**

Cilj istraživanja je da se ispitaju varijacije serumskog karcinoembrionskog antigena (CEA), citokeratin 19 fragmenta (CYFRA21-1) i antigena skvamoznih ćelija (SCC) kod pacijenata sa karcinomom pluća (KP) i njihov dijagnostički značaj u vezi sa ultrazvučno vođenom transbronhijalnom aspiracijom iglom (EBUS-TBNA).

**Metode:**

Ovo istraživanje je ispitivalo dijagnostički značaj testiranja tumorskih markera u serumu i zajedničko otkrivanje EBUS-TBNA za KP kod 150 pacijenata sa sumnjom na KP.

**Rezultati:**

U poređenju sa benignim pacijentima, nivoi CYFRA21-1, SCC i CEA u serumu kod KP su bili viši (P<0,05). Kod pacijenata sa karcinomom skvamoznih ćelija (LSCC), karcinomom malih ćelija pluća (SCLC) i adenokarcinomom pluća, adenokarcinom pluća je imao veći nivo CEA u serumu (P<0,05). Sa druge strane, pacijenti sa LSCC su imali više nivoe SCC i CYFRA21-1 u serumu (P<0,05). U poređenju sa svakim indeksom koji se detektuje pojedinačno, AUC kombinovanog otkrivanja svakog indeksa radi dijagnoze KP i identifikacije patoloških tipova KP je bio povećan.

**Zaključak:**

Pokazan je klinički značaj serumskih markera CYFRA21-1, SCC i CEA u vezi sa EBUS-TBNA u dijagnostičke svrhe i identifikaciju patoloških tipova KP

## Introduction

Lung cancer (LC) is a malignant tumor with a high mortality rate in China and a pyramidal increase in morbidity. Studies have manifested that early diagnosis and effective treatment can prolong their lives and improve their quality of life [1]. Endobronchial ultrasound-guided transbronchial needle aspiration (EBUS-TBNA) is a major diagnostic method for LC. However, its diagnostic accuracy is nearly proportional to the focus size. Tiny lesions are hard to sample and have an impact on diagnostic accuracy. In clinical operations, ultrasound highlights masses with abundant blood vessels for puncture, which is supposed to result in bleeding [2] [3]. Consequently, exploring an early diagnostic method with convenient operation and supernal repeatability is vital. In the past few years, tumor markers have been broadly applied in the clinical diagnosis and prognostic assessment of LC. Squamous cell carcinoma (SCC) antigen, cytokeratin fragment 19 (CYFRA21-1), and serum carcinoembryonic antigen (CEA) are nearly concerned in LC [4]. LC has no distinct specificity at its onset, but the relevant serum tumor markers are altered to varying degrees in patients with the disease [5], implying that LC is supposed to be diagnosed by testing variations of serum tumor markers. Accordingly, this study was to explore variations of CYFRA21-1, SCC, and CEA and the diagnostic value of EBUS-TBNA in patients with LC and offer a reference for the early diagnosis of the disease.

## Materials and methods

### Clinical data

From February 2018 to May 2021, 150 patients with suspected lung malignant lesions were selected. The LC group (n = 118) and the benign group (n = 32) were divided based on pathology and laboratory diagnosis. LC met the diagnostic criteria issued by the Chinese Medical Association Guidelines (Edition 2018) [6].

Inclusion criteria: patients with complete clinical data, patients with explicit pathological examination results, 18 years old or more.

Exclusion criteria: patients who received radiotherapy and chemotherapy prior to sampling, patients with other malignant tumor diseases, patients with a history of lung surgery, patients with severe liver and kidney system diseases, patients with respiratory malformations.

This study involved 68 cases of adenocarcinoma, 18 cases of small cell lung cancer (SCLC), and 11 cases of other types of cancer. No differences were shown in general data between the two groups (P>0.05), as presented in Table 1.

**Table 1:** Comparison of general data between the LC group and the benign group.

Classification	The LC<br>(n=118)	The benign<br>(n=32)	2/t	P
Gender: Male (cases)	79	12	0.080	0.778
Age (years)	63.72±10.19	59.90±8.12	1.957	0.052
Smoking history (cases)	34	9	0.006	0.939
Drinking history (cases)	27	5	0.790	0.374
Complicated with underlying<br>diseases (cases)	31	6	0.766	0.381
Systolic pressure (mmHg)	129.64 ± 10.30	128.13 ± 9.76	0.744	0.458
Diastolic blood pressure (mmHg)	86.19 ± 5.04	85.73 ± 5.19	0.455	0.650
Heart rate (times/min)	81.25 ± 4.07	80.71 ± 4.92	0.636	0.526
EBUS-TBNA (cases,%)	75(63.56)	27(86.49)	6.926	0.008

### Methods

Serum tumor marker test: After obtaining 5 mL fasting venous blood, centrifugation was carried out on a Beckman Coulter high-speed centrifuge, and the supernatant was collected. Serum CEA and CYFRA21-1 were tested by Roche E170 automatic chemiluminescence immunoassay analyzer and corresponding kits. The normal reference value for CEA is 0∼10 ng/mL and 0–3.3 ng/mL for CYFRA21-1. SCC was examined by an I2000SR chemiluminescence analyzer (Abbott, USA). Normal reference values are manifested in the instructions and relevant guidelines.

*EBUS-TBNA:* Before surgery, patients were fasting and water-deprived, and venous access was established after entering the operating room. Conventional electronic bronchoscopes were used for airway examination, and ultrasound bronchoscopes (Japan Olympus company, BF-UC260FW type) were inserted through the mouth or nose. According to CT results, EBUS-TBNA test was performed on the affected subsegment bronchi or enlarged lymph nodes. The specimens were collected using disposable needles or biopsy forceps (In some patients, endobronchial ultrasound with a guide sheath was used to collect specimens) and placed in a solution containing 10% formaldehyde.

### Observation indexes

(1) Serum CYFRA21-1, SCC, CEA, CYFRA21-1, and EBUS-TBNA test results in benign LC patients were compared to analyze the diagnostic value of the combined test of each indicator. (2) Serum CYFRA21-1, SCC, CEA, and EBUS-TBNA test results in patients with different pathological types of LC were compared to analyze the value of combined detection.

### Statistical processing

Data were processed using SPSS24.0 software, enumeration data were represented as percent, and the differences between groups were compared using χ^2^ test. Measurement data were shown as (x̄±s) after the normal test. Comparisons between groups were made using the t-test. The receiver operator characteristic (ROC) curve was utilized to analyze the value of serum CYFRA21-1, SCC, CEA, and EBUS-TBNA to diagnose LC and identify pathological types. AUC values were analyzed by the Z test. Significant differences were accepted at P<0.05.

## Results

### Comparison of serum CEA, SCC, CYFRA21-1 and EBUS-TBNA test results

**Figure 1:**
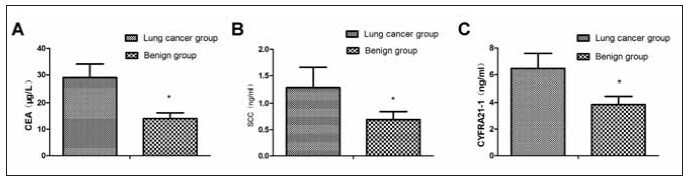
Comparing serum biomarker concentrations between the LC group and the benign group, (A) serum CEA levels, (B) serum SCC levels, (C) serum CYFRA21-1 levels. *P < 0.05.

Compared to the benign group, LC patients had higher levels of serum CEA, SCC, and CYFRA21-1 (*P*<0.05), as presented in Figure 1. EBUS-TBNA examination accuracy in LC was 65.25% (77/118), which was 84.38% (27/32) in the benign group (*P*<0.05).

### Diagnostic value of serum CEA, SCC, CYFRA21-1 conjugating with EBUS-TBNA in LC

**Figure 2:**
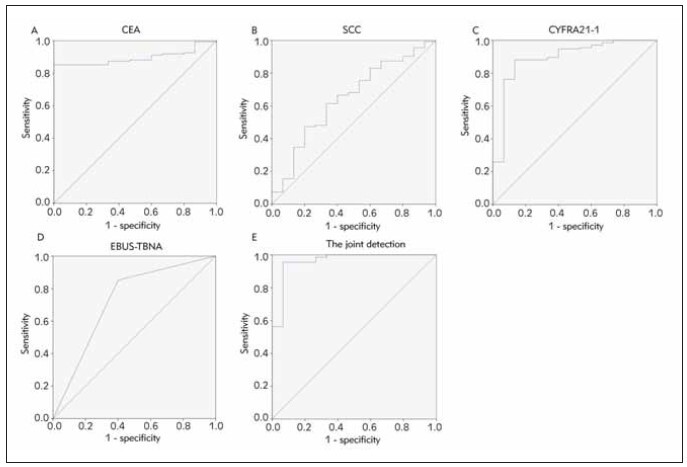
The diagnostic value of ROC curve analysis to discriminate the LC patients from the benign patients, (A) serum CEA levels, (B) serum SCC levels, (C) serum CYFRA21-1 levels, (D) EBUS-TBNA, (E) serum CEA, SCC, CYFRA21-1 combined with EBUS-TBNA.

We adopted the ROC curve and AUC values to assess the diagnosis value of the model. Although serum CEA, SCC, CYFRA21-1, and EBUS-TBNA each had diagnostic value for LC, the sensitivity and AUC of combined diagnosis were higher than that of single diagnosis, indicating a higher diagnostic value (*P*<0.05), as manifested in Table 2 and Figure 2.

**Table 2:** Analysis of serum CEA, SCC, CYFRA21-1 conjugating with EBUS-TBNA to test the diagnostic value for LC. Vs. the combined test, *P < 0.05.

Indexes	Cut-off values	AUC	SE	95% CI
CEA	21.06 μg/L	0.895*	0.026	0.845~0.946
SCC	0.95 ng/mL	0.645*	0.075	0.498~0.793
CYFRA21-1	5.07 ng/mL	0.891	0.049	0.796~0.987
EBUS-TBNA		0.726*	0.078	0.573~0.879
Combined detection		0.961	0.029	0.905~1.000

### Serum CEA, SCC, CYFRA21-1, and EBUS-TBNA results in patients with different types of LC

**Figure 3:**
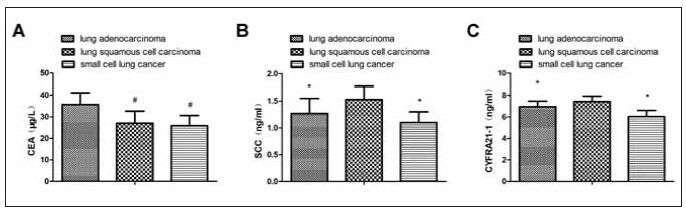
Comparing serum biomarker concentrations between the different pathological types of LC, (A) serum CEA levels, (B) serum SCC levels, (C) serum CYFRA21-1 levels. Vs. the lung adenocarcinoma, # *P* < 0.05; And vs. the LSCC, *P < 0.05.

In contrast to patients diagnosed with LSCC and SCLC, those with lung adenocarcinoma exhibited elevated levels of serum CEA. However, when compared to patients with lung adenocarcinoma and SCLC, individuals diagnosed with LSCC demonstrated higher concentrations of serum SCC and CYFRA21-1 (*P*<0.05), as presented in Figure 3. The diagnostic accuracy rate of EBUS-TBNA for lung adenocarcinoma, LSCC, and SCLC was 67.90% (55/81), 57.14% (8/14), and 61.90% (13/21), respectively, showing no statistical differences (*P*>0.05).

### Analysis of serum CEA, SCC, CYFRA21-1 conjugating with EBUS-TBNA to identify LSCC and lung adenocarcinoma

**Figure 4:**
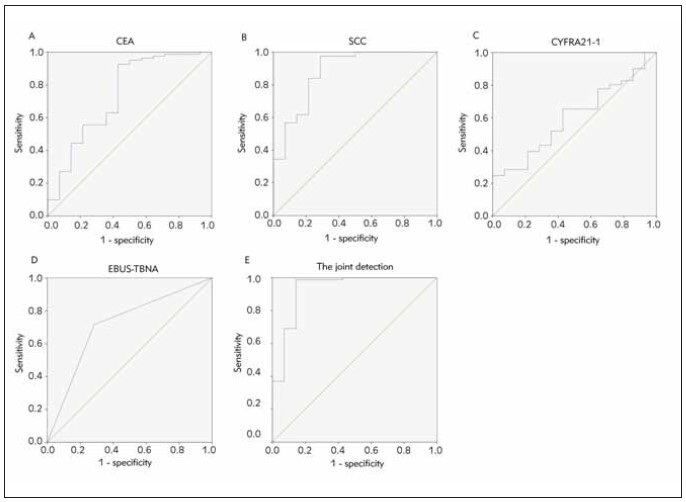
The diagnostic value of ROC curve analysis to discriminate LSCC patients from lung adenocarcinoma patients, (A) serum CEA levels, (B) serum SCC levels, (C) serum CYFRA21-1 levels, (D) EBUS-TBNA, (E) serum CEA, SCC, CYFRA21-1 combined with EBUS-TBNA.

The diagnostic efficacy of serum biomarkers and EBUS-TBNA in LSCC and lung adenocarcinoma was assessed using ROC curves and AUC analysis. The findings indicated that the combined diagnosis of serum CEA, SCC, CYFRA21-1, and EBUS-TBNA had a higher diagnostic value (AUC=0.929) compared to the individual diagnostic approach (*P*<0.05), as presented in Table 3 and Figure 4.

**Table 3:** Analysis of serum CEA, SCC, CYFRA21-1 conjugating with EBUS-TBNA to examine the identified value of LSCC and lung adenocarcinoma. Vs. the combined test, *P < 0.05.

Indexes	Cut-off values	AUC	SE	95% CI
CEA	29.16 μg/L	0.738	0.083	0.576∼0.900
SCC	1.39 ng/mL	0.878	0.057	0.768∼0.989
CYFRA21-1	7.06 ng/mL	0.602	0.073	0.459∼0.746
EBUS-TBNA		0.715	0.076	0.566∼0.864
Combined detection		0.929	0.047	0.837∼1.000

### Analysis of serum CEA, SCC, CYFRA21-1 conjugating with EBUS-TBNA to identify LSCC and lung adenocarcinoma

**Figure 5:**
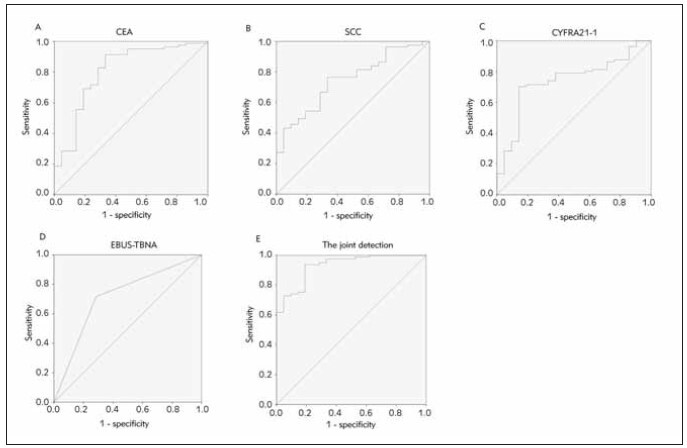
The diagnostic value of ROC curve analysis to discriminate LSCC patients from SCLC patients, (A) serum CEA levels, (B) serum SCC levels, (C) serum CYFRA21-1 levels, (D) EBUS-TBNA, (E) serum CEA, SCC CYFRA21-1 combined with EBUS-TBNA.

Next, we evaluated the diagnostic value of serum biomarkers and EBUS-TBNA for LSCC and SCLC. The results suggested that the combined diagnosis of serum CEA, SCC, CYFRA21-1, and EBUS-TBNA had a higher diagnostic value (AUC=0.931) compared to the individual diagnostic approach (*P*<0.05), as shown in Table 4 and Figure 5.

**Table 4:** Analysis of serum CEA, SCC, CYFRA21-1 combined with EBUS-TBNA to examine the identified value of LSCC and SCLC. Vs. the combined test, *P < 0.05.

Indexes	Cut-off values	AUC	SE	95% CI
CEA	30.51 μg/L	0.805*	0.059	0.689∼0.921
SCC	1.19 ng/mL	0.745*	0.055	0.637∼0.853
CYFRA21-1	6.54 ng/mL	0.740*	0.058	0.626∼0.853
EBUS-TBNA		0.715*	0.064	0.589∼0.841
Combined detection		0.931	0.028	0.876∼0.986

### Analysis of serum CEA, SCC, CYFRA21-1 conjugatingwith EBUS-TBNA to identify lung adenocarcinoma and SCLC

**Figure 6:**
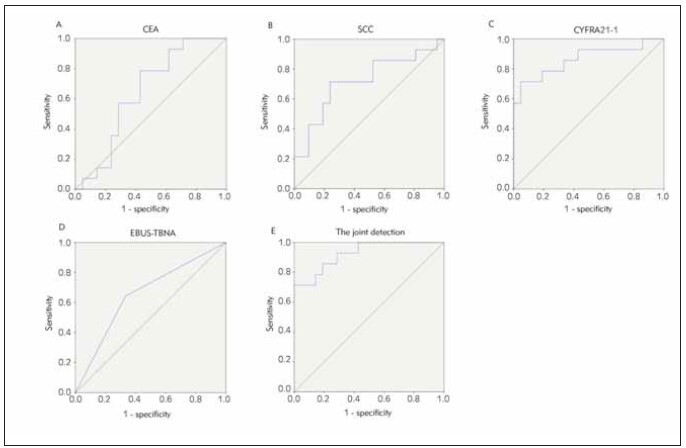
The diagnostic value of ROC curve analysis to discriminate lung adenocarcinoma patients from SCLC patients, (A) serum CEA levels, (B) serum SCC levels, (C) serum.

Finally, we also evaluated the diagnostic value of these serum biomarkers and EBUS-TBNA for lung adenocarcinoma and SCLC. The results were following what we expected. The combination of serum biomarkers and EBUS-TBNA had a higher diagnostic value (AUC=0.925) (*P*<0.05), as shown in Table 5 and Figure 6.

**Table 5:** Analysis of serum CEA, SCC, CYFRA21-1 conjugating with EBUS-TBNA to examine the identified value of lung adenocarcinoma and SCLC. Vs. the combined test, *P < 0.05.

Indexes	Cut-off indexes	AUC	SE	95% CI
CEA	5.39 μg/L	0.643	0.093	0.460∼0.826
SCC	0.82 ng/mL	0.718	0.093	0.535∼0.901
CYFRA21-1	3.05 ng/mL	0.864	0.070	0.727∼0.956
EBUS-TBNA		0.655	0.096	0.466∼0.843
Combined detection		0.925	0.044	0.840∼0.998

## Discussion

In cancer cells, tumor markers are substances released into body fluids or tissues characteristic of malignant tumors [7]. CEA and CYFRA21-1 are nearly associated with the occurrence of multiple malignant tumors. CEA is a tumor-associated antigen extracted from colon cancer and embryonic tissues. It is an acid glycoprotein with a specific human embryonic antigen on the cancer cell surface [8]. The CYFRA21-1 polypeptide is found primarily in lung tumor epithelial cytoplasm, and it can either be degraded by proteases or released into the blood in the form of dissolved fragments after cell death [8]. Relevant studies have testified that CYFRA21-1 has high sensitivity and specificity in diagnosing LSCC [9] [10]. SCC is an antigenic element separated from cervical squamous cells. Furthermore, serum SCC in patients with SCC is distinctly elevated [11] [12]. The results indicated that serum CYFRA21-1, SCC, and CEA in the LC group were elevated versus in the benign group.

A growing number of Chinese patients are diagnosed with LC each year. Still, no typical clinical symptoms and manifestations in the early stages lead to a diagnosis delay. Hence, early detection and treatment of LC are critical [13] [14]. EBUS-TBNA involves transbronchial transmural needle aspiration guided by ultrasound bronchoscope for puncture sampling as a prevalent method of lung disease examination. Nevertheless, this method has limitations; for instance, when the focus diameter is small, it will be challenging to position and clip the tissue accurately, which will result in missed diagnoses and misdiagnosis [15]. The study manifested that the diagnostic accuracy of EBUS-TBNA examination for LC is 60.67%, which is slightly lower than the result of a previous study [16]. Meanwhile, it is supposed to be associated with the population size in this study. Therefore, the sample size should be expanded for further analysis. Serum tumor markers of patients with LC were elevated versus patients with benign lung diseases. Therefore, the author claims that EBUS-TBNA combined diagnosis should improve diagnostic accuracy by detecting serum tumor markers. Additionally, the AUC of serum CYFRA21-1, SCC, and CEA conjugating with EBUS-TBNA to diagnose LC was augmented compared to single detection.

SCLC, LSCC, and lung adenocarcinoma are prevalent pathological types of LC. Adenocarcinoma is abundant in blood vessels and prone to local invasion and hematological metastasis. In SCC, the progression is dilatory, and late metastasis occurs. After surgery, most SCC patients have a good prognosis. As the most malignant LC, SCLC progresses rapidly and invades more deeply. It is uncertain when distant metastases will occur, and surgical treatment has a low probability [17] [18]. Identifying the pathological types of LC early to implement later therapeutic measures is beneficial. The results elucidated that serum CEA of patients with lung adenocarcinoma was augmented versus patients with LSCC and SCLC, and serum SCC and CYFRA21-1 in patients with LSCC were elevated versus patients with lung adenocarcinoma and SCLC. It is primarily linked to: CEA is mainly released from epithelial tumors, LC originates from the bronchial mucosal epithelium, and SCC is an antigen generated and secreted by LSCC [19] [20]. In the meantime, a relevant report also declares that CYFRA21-1 in patients with LSCC is elevated versus patients with lung adenocarcinoma [21]. Furthermore, it is feasible to distinguish the pathological type of LC by examining serum markers. Nonetheless, corresponding reports have clarified that the specificity of monitoring tumor markers alone to distinguish different pathological types of LC is reduced [22] [23]. Accordingly, this study used a combination of serum indices and EBUS-TBNA examination for diagnosis. The results elucidated that AUC of serum CYFRA21-1, SCC, and CEA conjugating with EBUS-TBNA to distinguish SCLC, lung adenocarcinoma, and LSCC was elevated versus alone detection of each index, manifesting that combined test was valuable to identify pathological types of LC.

In short, patients with LC show increased serum CYFRA21-1, SCC, and CEA, and joint test of serum tumor marker and EBUS-TBNA examination has diagnostic value for LC and can distinguish pathological types of LC.

## Dodatak

### Acknowledgments

Not applicable.

### Funding

Not applicable.

### Data availability

The article includes the figures and tables used to support this study’s findings.

### Authors’ contributions

YanJia Du designed the research study. Ya Wen performed the research. JieYu Huang provided help and advice on the experiments. YanJia Du analyzed the data. YanJia Du, Ya Wen, and JieYu Huang wrote the manuscript. All authors contributed to editorial changes in the manuscript. All authors read and approved the final manuscript.

### Conflict of interest statement

All the authors declare that they have no conflict of interest in this work.

## References

[1] O'Rourke K (2022). Lung cancer screening associated with earlier diagnosis and improved survival. Cancer.

[2] Wang S, Ma P, Ma G, Lv Z, Wu F, Guo M, et al (2020). Value of serum tumor markers for predicting EGFR mutations and positive ALK expression in 1089 Chinese non-small-cell lung cancer patients: A retrospective analysis. Eur J Cancer.

[3] Figueiredo V R, Cardoso P F G, Jacomelli M, Santos L M, Minata M, Terra R M (2020). EBUS-TBNA versus surgical mediastinoscopy for mediastinal lymph node staging in potentially operable non-small cell lung cancer: A systematic review and meta-analysis. J Bras Pneumol.

[4] Yuan J, Sun Y, Wang K, Wang Z, Li D, Fan M, et al (2022). Development and validation of reassigned CEA,CYFRA21-1 and NSE-based models for lung cancer diagnosis and prognosis prediction. BMC Cancer.

[5] Nocini R, Sanchis-Gomar F, Lippi G, Mattiuzzi C (2023). Red blood cell distribution width (RDW) is a significant predictor of survival in laryngeal cancer patients: Systematic literature review and meta-analysis. J Med Biochem.

[6] 6. *** (2018). Chinese Medical Association guidelines for clinical diagnosis and treatment of lung cancer (Edition 2018). Zhonghua Zhong Liu Za Zhi.

[7] Chouaid C, Salaün M, Gounant V, Febvre M, Vergnon J M, Jouniaux V, et al (2019). Clinical efficacy and cost-effectiveness of endobronchial ultrasound-guided transbronchial needle aspiration for preoperative staging of non-small-cell lung cancer: Results of a French prospective multicenter trial (EVIEPEB). PLoS One.

[8] Vollrath Jt, Schindler Cr, Herrmann E, Verboket Rd, Henrich D, Marzi I, et al (2023). Evaluation of Cyfra 21-1, Angiopoetin-2, Pentraxin-3, Srage, Il-6, and Il-10 in polytraumatized patients with concomitant thoracic trauma-helpful markers to predict pneumonia?. Shock.

[9] Kotowicz B, Fuksiewicz M, Jonska-Gmyrek J, Bidzinski M, Kowalska M (2016). The assessment of the prognostic value of tumor markers and cytokines as SCCAg, CYFRA 21.1, IL-6, VEGF and sTNF receptors in patients with squamous cell cervical cancer, particularly with early stage of the disease. Tumour Biol.

[10] Yang Y, Huang X, Zhou L, Deng T, Ning T, Liu R, et al (2019). Clinical use of tumor biomarkers in prediction for prognosis and chemotherapeutic effect in esophageal squamous cell carcinoma. BMC Cancer.

[11] Zhao W, Liu Y, Kong F, Li Y (2020). Correlations of pathological stage and prognosis of NSCLC patients with changes in serum CEA and CYFRA 21-1 and CT characteristics. Panminerva Med.

[12] Zhu K, Chen L, He C, Lang Y, Kong X, Qu C, et al (2020). Prediction of pleural invasion in challenging non-small-cell lung cancer patients using serum and imaging markers. Dis Markers.

[13] Jiang C, Zhao M, Hou S, Hu X, Huang J, Wang H, et al (2022). The indicative value of serum tumor markers for metastasis and stage of non-small cell lung cancer. Cancers (Basel).

[14] Qu T, Zhang J, Xu N, Liu B, Li M, Liu A, et al (2019). Diagnostic value analysis of combined detection of Trx, CYFRA21-1 and SCCA in lung cancer. Oncol Lett.

[15] Martin-Deleon R, Solarat B, Moisés J, Lucena C M, Fontana A, Marrades R M, et al (2022). EBUS-TBNA in extrathoracic malignancies: Diagnostic and prognostic implications. Lung.

[16] Jia K, Li W, Wang F, Qu H, Qiao Y, Zhou L, et al (2016). Novel circulating peptide biomarkers for esophageal squamous cell carcinoma revealed by a magnetic beadbased MALDI-TOFMS assay. Oncotarget.

[17] Muley T, Rolny V, He Y, Wehnl B, Escherich A, Warth A, et al (2018). The combination of the blood based tumor biomarkers cytokeratin 19 fragments (CYFRA 21-1) and carcinoembryonic antigen (CEA) as a potential predictor of benefit from adjuvant chemotherapy in early stage squamous cell carcinoma of the lung (SCC). Lung Cancer.

[18] Dal Bello M G, Filiberti R A, Alama A, Orengo A M, Mussap M, Coco S, et al (2019). The role of CEA, CYFRA21-1 and NSE in monitoring tumor response to nivolumab in advanced non-small cell lung cancer (NSCLC) patients. J Transl Med.

[19] Bi H, Yin L, Fang W, Song S, Wu S, Shen J (2023). Association of CEA, NSE, CYFRA 21-1, SCC-Ag, and ProGRP with clinicopathological characteristics and chemo therapeutic outcomes of lung cancer. Lab Med.

[20] Dong Z, Li H, Jiang H, Wu C (2017). Evaluation of cytology in lung cancer diagnosis based on EBUS-TBNA. J Cytol.

[21] Singh R, Lal A J L C (2019). EBUS-TBNA for the diagnosis of lung parenchymal lesions.

[22] Righi L, Franzi F, Montarolo F, Gatti G, Bongiovanni M, Sessa F, et al (2017). Endobronchial ultrasound-guided transbronchial needle aspiration (EBUS-TBNA)-from morphology to molecular testing. J Thorac Dis.

[23] Jiang M, Chen P, Guo X, Zhang X, Gao Q, Zhang J, et al (2023). Identification of EGFR mutation status in male patients with non-small-cell lung cancer: Role of (18)F-FDG PET/CT and serum tumor markers CYFRA21-1 and SCC-Ag. EJNMMI Res.

